# Unusual Sign from an Unusual Cause: Wellens' Syndrome due to Myocardial Bridging

**DOI:** 10.1155/2018/3105653

**Published:** 2018-07-25

**Authors:** Paurush Ambesh, Dikshya Sharma, Aditya Kapoor, Aviva-Tobin Hess, Vijay Shetty, Gerald Hollander, Jacob Shani, Stephan Kamholz, Arjun Saradna, Isaac Akkad, Chukwudi Obiagwu

**Affiliations:** ^1^Department of Internal Medicine, Maimonides Medical Center, New York City, NY, USA; ^2^Department of Cardiology, Sanjay Gandhi Post Graduate Institute of Medical Sciences, Lucknow, India; ^3^Department of Cardiology, Maimonides Medical Center, New York City, NY, USA

## Abstract

It is vital to recognize correctly, chest pain of cardiac etiology. Most commonly, it is because of blood supply-demand inequity in the myocardium. However, the phenomenon of myocardial bridging as a cause of cardiac chest pain has come to attention reasonably recently. Herein, a coronary artery with a normal epicardial orientation develops a transient myocardial course. If the cardiac muscle burden is substantial, the respective artery gets compressed during each cycle of systole, thereby impeding blood flow in the artery. Hence, myocardial bridging has been attributed to as a rare cause of angina. In this case report, the authors discuss a patient in whom myocardial bridging turned out to be an elusive cause of angina. We wish to underscore the importance of being clinically mindful of myocardial bridging when assessing a patient with angina.

## 1. Introduction

Chest pain of cardiac origin is commonly ischemic in etiology. However, the phenomenon of myocardial bridging as a cause of chest pain has come to attention recently. Herein, an epicardial coronary artery develops a transient myocardial course. During systole, a portion of the vessel is mechanically compressed and results in ischemia. This has been attributed to as a rare cause of angina. Here, we present a patient who was diagnosed with the same.

## 2. Case Presentation

**Figure 1:**
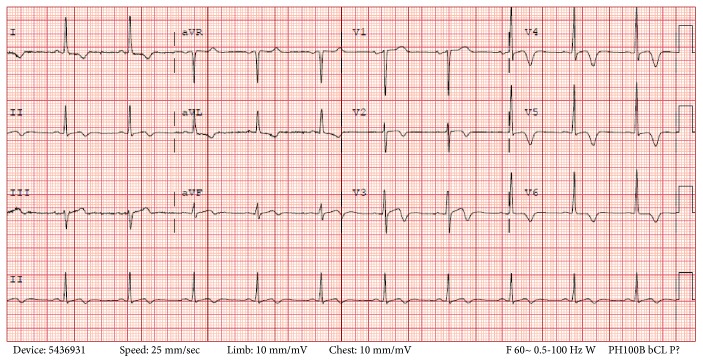
12-lead EKG showing Wellens sign in leads V2,V3.

A 51-year-old man with no past medical history presented to the emergency room with pressure-like chest pain of two-day duration. He had multiple episodes of pain, and each lasted for around 20 minutes. It was associated with palpitations and exacerbated by physical exertion. He was a former smoker and reportedly quit smoking 20 years back. His blood pressure was 153/95 mmHg, temperature 98.1°F, heart rate 73/min regular, and respiratory rate 18/min. Electrocardiogram (ECG) showed Type 1 Wellens' Biphasic pattern in leads V2 and V3. (Figure 1).

Three sets of cardiac troponins were normal. Wellens' pattern on ECG is highly specific (89%) for critical left anterior descending artery (LAD) stenosis [1, 2]. Therefore, the patient was sent for emergent cardiac catheterization. Coronary angiography revealed normal left main, left circumflex, and right coronary artery. However, LAD artery showed moderate myocardial bridging.

## 3. Discussion

In myocardial bridging, a coronary artery that runs typically on the epicardium develops a transient intramyocardial course. This section of the artery gets compressed during ventricular systole. Generally asymptomatic, myocardial bridging may cause angina, arrhythmias (supraventricular and ventricular tachycardia), myocardial ischemia or infarction, and even sudden cardiac death. The degree of ischemia has been shown to correlate with the degree of systolic compression directly.

On coronary angiography, most myocardial bridging is predominantly found to occur in the middle portion of the LAD artery. However, on autopsy, the right coronary and left circumflex arteries have shown bridging at comparable rates [3].

Angiographically detected rates of bridging have been reported to be 0.5–12%, with increased detection using provocative testing [4]. The proximal portion of the bridged segment is prone to accelerated atherosclerosis.

Hypertrophic obstructive cardiomyopathy (HOCM) has shown increased association with myocardial bridging [5]. Orthotopic heart transplant recipients also show increased prevalence of myocardial bridging [6]. This occurs due to greater myocardial hypertrophy and stiffness after transplant, which in turn causes more systolic vessel compression.

First-line management for myocardial bridging utilizes medical treatment with beta blockers and nondihydropyridine calcium channel blockers. Therapeutic benefit results from decreased chronotropy and inotropy. This subsequently causes diastolic prolongation. Nitrates cause secondary tachycardia, with reflex sympathetic stimulation, and therefore are contraindicated. In cases refractory to medical management, surgical myotomy, coronary artery stenting, or coronary artery bypass grafting (CABG) can be pursued.

Coronary stenting has been tried but is associated with serious complications like coronary artery perforation and stent fracture. Surgical myotomy may result in right ventricle dissection in patients with deep subendocardial myocardial bridges [7]. Our patient was started on aspirin, amlodipine, metoprolol, and rosuvastatin. His hospital course was uneventful, and he was discharged on pharmacological therapy. He continues to follow up in the cardiology clinic, and his symptoms are well controlled.

## Supplementary Materials

Supplementary Materials Supplementary Video: coronary angiogram shows compression of proximal left anterior descending artery with each systole.
